# Effect of CAD/CAM milling protocols on surface topography and mechanical behavior of polycrystalline zirconia

**DOI:** 10.1016/j.dental.2025.11.004

**Published:** 2025-11-08

**Authors:** Laura Carolina Kepler, Paula Benetti, Vitor Trancoso Britto, Alvaro Della Bona, Yu Zhang, Marcia Borba

**Affiliations:** a Postgraduate Program in Dentistry, School of Dentistry, University of Passo Fundo, Passo Fundo, RS, Brazil; b Department of Preventive and Restorative Sciences, School of Dental Medicine, University of Pennsylvania, Philadelphia, PA, USA; c Division of Dentistry, School of Medical Sciences, University of Manchester, Manchester, United Kingdom

**Keywords:** Ceramic, Zirconia, CAD/CAM, Flexure strength

## Abstract

**Objectives:**

Even though improvements in CAD/CAM technology have allowed the milling protocol to be modified according to specific treatment requirements, it remains unclear how different protocols affect the surface quality and mechanical properties of the final restoration. This study aims to evaluate the effect of the CAD/CAM milling protocol on the topography, flexural strength, and reliability of 3 mol% yttria partially stabilized zirconia (3Y-PSZ).

**Methods:**

Disc-shaped specimens (∅12 mm×1.2 mm) were fabricated using three different CAD/CAM dry-milling protocols (n = 20): slow (S), normal (N), and fast (F). Control polished specimens were fabricated by cutting pre-sintered CAD/CAM blocks using a cutting machine and polishing with silicon carbide papers, in wet (P_wet_) and dry (P_dry_) conditions (n = 20). Surface topography was evaluated using an optical profilometer (n = 3). Specimens were subjected to a piston-on-three-balls flexural strength test using a universal testing machine. Fractographic analysis was performed using optical and scanning electron microscopes. The characteristic flexural strength (σ_0_), Weibull modulus (*m*), and the 90 % confidence intervals (90 % CI) were estimated. XRD was used to identify the tetragonal, cubic, and monoclinic phases.

**Results:**

Distinct topographies were observed for polished and CAD/CAM-milled specimens. Surface roughness parameters (Sa and Sz) followed a decreasing order: F > N > S > P (P_wet_ and P_dry_) There was no difference for σ_0_ and *m* values among the three CAD/CAM milling protocols. P_dry_ had similar σ_0_ and *m* to CAD/CAM-milled groups, while P_wet_ resulted in lower values.

**Significance:**

Distinct CAM-milling protocols produce apparent differences in the topography of 3Y-PSZ while preserving flexural strength and reliability.

## 1. Introduction

Zirconia polycrystals are widely used in Dentistry due to their superior mechanical properties. 3 mol% yttria-stabilized tetragonal zirconia polycrystal (3Y-TZP) has flexural strength values varying from 900 to 1300 MPa and fracture toughness between 3.5 and 6.0 MPa m^½^, depending on the composition and surface treatments [1-4]. These materials are also highly valued for their excellent biocompatibility and corrosion resistance [3]. 3Y-TZP benefits from a phase-transformation toughening mechanism, where, when subjected to stresses, a tetragonal-to-monoclinic crystal phase transformation is induced. This results in a 3–4 % expansion in the crystal volume at the crack tip, generating compressive stresses that oppose crack propagation [2-4]. Therefore, 3Y-TZP is used in Dentistry for a wide range of clinical applications, including infrastructures for single crowns up to complex long span fixed partial dentures (FPD) [2].

Besides the good mechanical properties of 3Y-TZP, modifications on the material composition and microstructure were proposed aiming to enhance their optical properties. One strategy was to reduce the content of alumina sintering additive and increase sintering temperatures. This approach increases the material’s density and grain size [2,4-6], while reducing light scattering centers [7]. This esthetically improved zirconia is called 3 mol% yttria partially stabilized zirconia (3Y-PSZ) due to the presence of a small amount of cubic content (<30 %) as a result of high-temperature sintering [5,8]. 3Y-PSZ has improved appearance, while preserving the mechanical properties of first generation 3Y-TZP, which allows the production of monolithic crowns and FPDs [2,5,8].

Polycrystalline zirconia restorations are usually produced with the CAD/CAM (computer-aided design/ computer-aided manufacturing) technology through subtractive manufacturing of partially or fully sintered blanks [2,4,9]. Recent improvements in the CAD/CAM technology allow the dentist and laboratory technician to select the milling protocol based on treatment requirements. Protocols range from a faster option, in which the restoration can be milled in just a few minutes, to a slower one that allows the production of refined anatomic features [9,10]. Although this novel approach has increased the efficiency of the production process and enabled a more patient-centered approach, it is not clear how the different protocols affect the surface characteristics and mechanical properties of the final restoration. Subtractive manufacturing of ceramics may introduce surface and sub-surface defects that could compromise their mechanical integrity and affect the restoration longevity [11-14].

Additionally, CAD/CAM milling can be performed in dry or wet conditions [15]. Wet milling involves the use of liquids, typically water, to control the heat during processing. Water helps remove debris from the cutting process, minimizing the adhesion of particles to the zirconia surface. Nevertheless, wet processing should not be performed with water impregnated with residues from different CAD/CAM materials as it may alter the properties of the final restoration [16]. Dry milling generates zirconia dust during the process and requires the use of special burs in conjunction with a vacuum pump. Findings in the literature are controversial regarding the effects of dry/wet processing on the mechanical and optical properties of zirconia [15-17].

Therefore, the objective of this study was to evaluate the effect of different CAD/CAM milling protocols on the surface topography and mechanical behavior of 3 mol% yttria-partially stabilized zirconia (3Y-PSZ). The study null hypotheses are: (1) the CAD/CAM milling protocol does not affect the surface topography, flexural strength, and Weibull modulus of 3Y-PSZ specimens; and (2) 3Y-PSZ polished specimens produced under dry and wet conditions have similar mechanical behavior.

## 2. Materials and methods

A 3 mol% yttria partially stabilized zirconia (3Y-PSZ, Zolid ZI, Amann Girrbach AG) was investigated. Five experimental groups were analyzed as described in Table 1. Three groups were produced using different CAD/CAM dry-milling protocols (n = 20): slow (S), normal (N), and fast (F). Two polished groups were fabricated by cutting pre-sintered CAD/CAM blocks and polishing with silicon carbide papers, in wet (P_wet_) and dry (P_dry_) conditions (n = 20). Qualitative and quantitative surface analysis was performed using an optical profilometer. Mechanical properties were assessed using a piston-on-three balls flexural strength test and fractographic analysis. X-ray diffraction (XRD) was used to identify the tetragonal, cubic, and monoclinic crystal phases.

### 2.1. Specimen fabrication

**Fig. 1.**
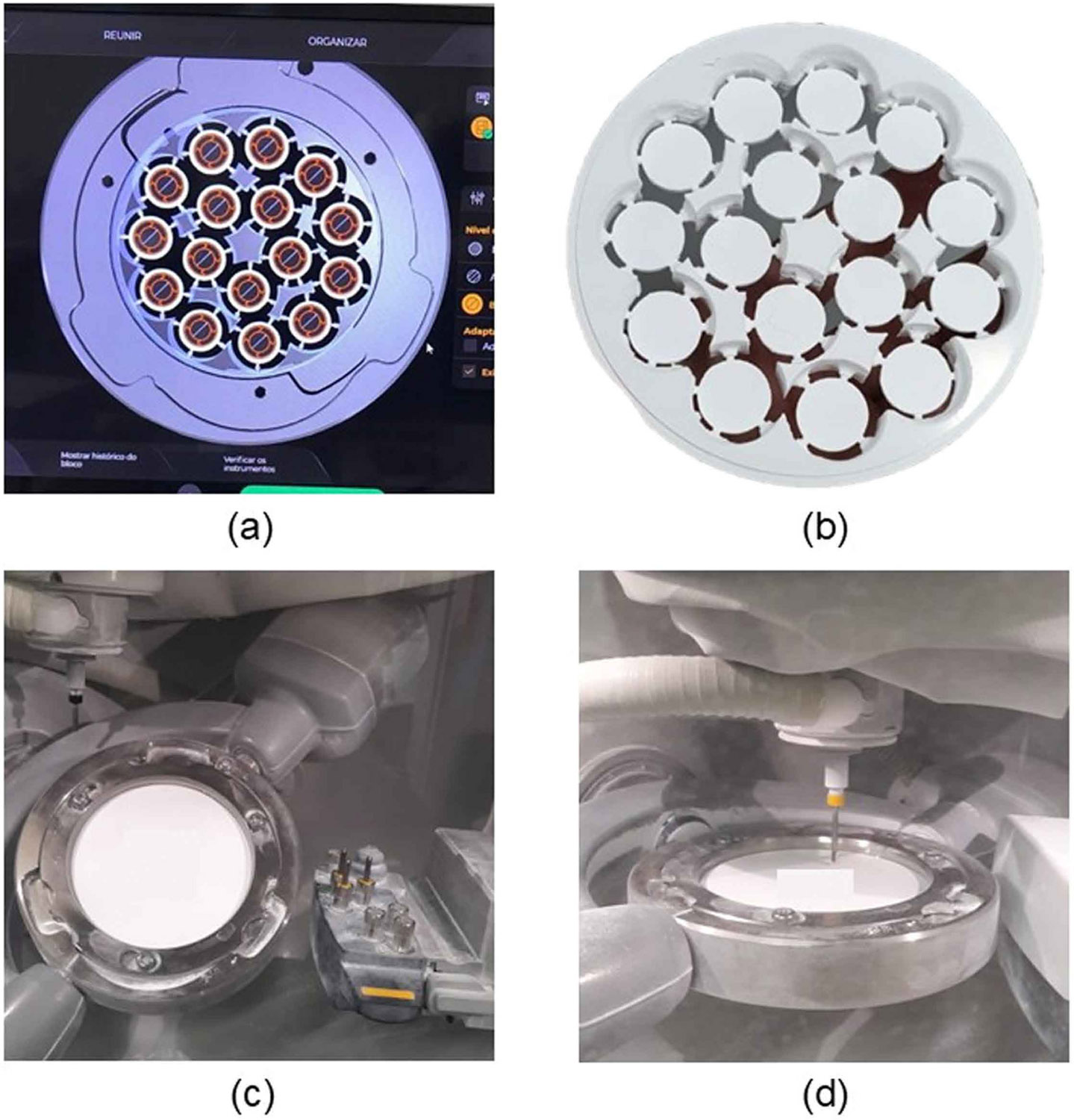
CAD/CAM processing: (a) specimen design in CAM 19.0 software; (b) pre-sintered zirconia blank after CAD/CAM milling, showing the disc-shaped specimens; (c) zirconia blank and diamond-coated burs placed in the In Lab MC X5 unit; (d) CAD/CAM dry-milling the zirconia specimens.

Disc-shaped specimens were produced with 1.2 mm in thickness and 12 mm in diameter. For the CAD/CAM milled groups, first a disc-shaped design was created in SolidWorks software and converted into a STL file. The file was imported to the CAM 19.0 software (Dentsply Sirona, Bensheim, Germany). Pre-sintered zirconia discs (3Y- Zolid ZI, Amann Girrbach AG, Koblach, Austria, dimensions of ∅ 98 mm×14 mm) were dry-milled using an InLab MC X5 unit (Dentsply Sirona, Bensheim, Germany) with three different protocols, normal, fast, and slow (Fig. 1). The same three types of diamond-coated burs for zirconium oxide were used for the three protocols: Bur 2.5 ZrO_2_ DC, Bur 1.0 ZrO_2_ DC, and Bur 0.5 ZrO_2_ DC (Dentisply Sirona, Benshiem, Germany). A total of four CAD/CAM blocks were used to produce the specimens. Each block was sub-divided in three areas, where 5–6 disc-shaped specimens were milled with each protocol (S, N, and F). The main difference between protocols is the burs` path and the time required to produce the specimens. Milling time followed a decreasing order: S>N > F.

For the polished groups, the 3Y pre-sintered zirconia discs were cut into blocks (17.0 mm×17.0 mm×12.0 mm) using a metallographic cutting machine (LCD 1200RPM, Biopdi, São Carlos/SP, Brazil) and a diamond blade. The blocks were then machined into cylinders (∅14.8 mm×12.0 mm) using a mechanical lathe (ROMI T240, ROMI S. A, Santa Barbara d’Oeste, SP, Brazil). Next, each cylinder was sectioned into disc-shaped specimens using the cutting machine with a dimension of 1.4 mm thickness and 14.8 mm diameter to compensate for the 23.37 % sintering shrinkage. One surface was polished with silicon carbide papers (sequence: #1000, #1200, #1500, #2000, and #2500) (Hermes Schleifmittel GmbH, Hamburg, Germany). For the wet group (P_wet_), cutting and polishing were performed under water cooling, while for the dry group (P_dry_) all steps were performed under dry conditions.

All specimens were sintered in a high-temperature furnace (Inlab Profire, Dentsply Sirona, Bensheim, Germany) following the manufacturer’s recommendations. In the heating phase, the temperature is increased from room temperature to a final temperature of 1450°C at a rate of 5–10 °C/min, and the final temperature is held for 2 h. The cooling process takes approximately 5 h, when the temperature is reduced from 1450°C to room temperature at a rate of 5 °C/min. No surface treatment was performed after sintering in any of the experimental groups.

### 2.2. Topography analysis

Qualitative and quantitative surface topographical analysis was performed using an optical profilometer (Profilm 3D, Filmetrics) equipped with 10x and 50x objective lens (Nikon) (n = 3). An area of 2 mm× 2 mm was scanned. The following parameters were obtained: Sa - arithmetic mean roughness; and Sz – maximum height.

The surface topography of the specimens was also analyzed using scanning electron microscope (SEM; VEGA3, Tescan Co., Ltd., Brno, Czech Republic).

### 2.3. Mechanical analysis

The biaxial flexure strength test was performed using a piston-on-three-balls setup in a universal testing machine (Instron), with a cross-head speed of 0.5 mm/min (n = 20). Tests were performed in distilled water at 37°C. A polyester strip was placed between the specimen and the piston to allow for a more homogeneous stress distribution. The biaxial flexural strength was calculated following ISO/FDIS 6872:2015 [18].

$$\sigma =-0.2387\;P(X-Y)∕b^{2}\;(A)\;X=(1+\nu )\;In\;{\left(r_{2}∕r_{3}\right)}^{2}+[\left(1-\nu \right)∕2]\;{\left(r_{2}∕r_{3}\right)}^{2}\;(B)\;Y=(1+\nu )\;[1+In\;{\left(r_{1}∕r_{3}\right)}^{2}]+(1-\nu )\;{\left(r_{1}∕r_{3}\right)}^{2}\;(C)$$

where P is the fracture load (N), v is Poisson’s ratio (0.32) [1], *r*_1_ is the radius of the support circle formed by the three balls (4.24 mm), *r*_2_ is the radius of the piston (0.78 mm), *r*_3_ is the radius of the specimen (mm), and b is the thickness of the specimen (mm).

The fracture surfaces of the specimens were analyzed using a scanning electron microscope (SEM; Mira3, Tescan Co., Ltd., Brno, Czech Republic), following standard fractographic principles [19]. To provide electrical conductivity, specimens were cleaned and sputter-coated with gold–palladium (Au-Pd) before SEM analysis using a secondary electron detector (SE, 5.0 kV).

The flexural strength data were analyzed using a two-parameter Weibull analysis and the Maximum Likelihood Estimate (MLE) to calculate the characteristic strength (σ_0_) and the Weibull modulus (*m*). The 90 % confidence intervals (90 % CI) were calculated using the Likelihood Ratio method. Statistical analysis was conducted using a reliability software (Weibull++, Reliasoft). The Chi-square test and Bonferroni-adjusted 90 % simultaneous confidence intervals were used for multiple pairwise comparisons of the Weibull modulus (Minitab Release 14, Minitab Inc). 90 % CI were chosen for the analyses to increase statistical power, especially considering that Bonferroni adjustments are highly conservative, and wider intervals can mask meaningful effects. A sample size of 20 was selected in accordance with the recommendations for the Weibull analysis [18].

### 2.4. D. Crystal phase identification

X-ray diffraction (XRD, X-ray Diffractometer, Rigaku, Tokyo, Japan) was used to identify monoclinic, tetragonal, and cubic crystal phases. The following parameters were applied: 2θ initial = 25; 2θ final = 80; step = 0.02; time per step = 0.50 s; current = 10 mA; voltage = 30 kV; KaCu = 1.54184. The crystalline phases were identified by comparing the diffraction patterns to reference data from the PDF-2 database (International Centre for Diffraction Data – ICDD). Semi-quantitative analysis of the relative amounts of each crystalline phase were determined by calculating the area under each diffraction peak, based on its intensity and width, using DIFFRAC.EVA software.

## 3. Results

### 3.1. Topography analysis

**Fig. 2.**
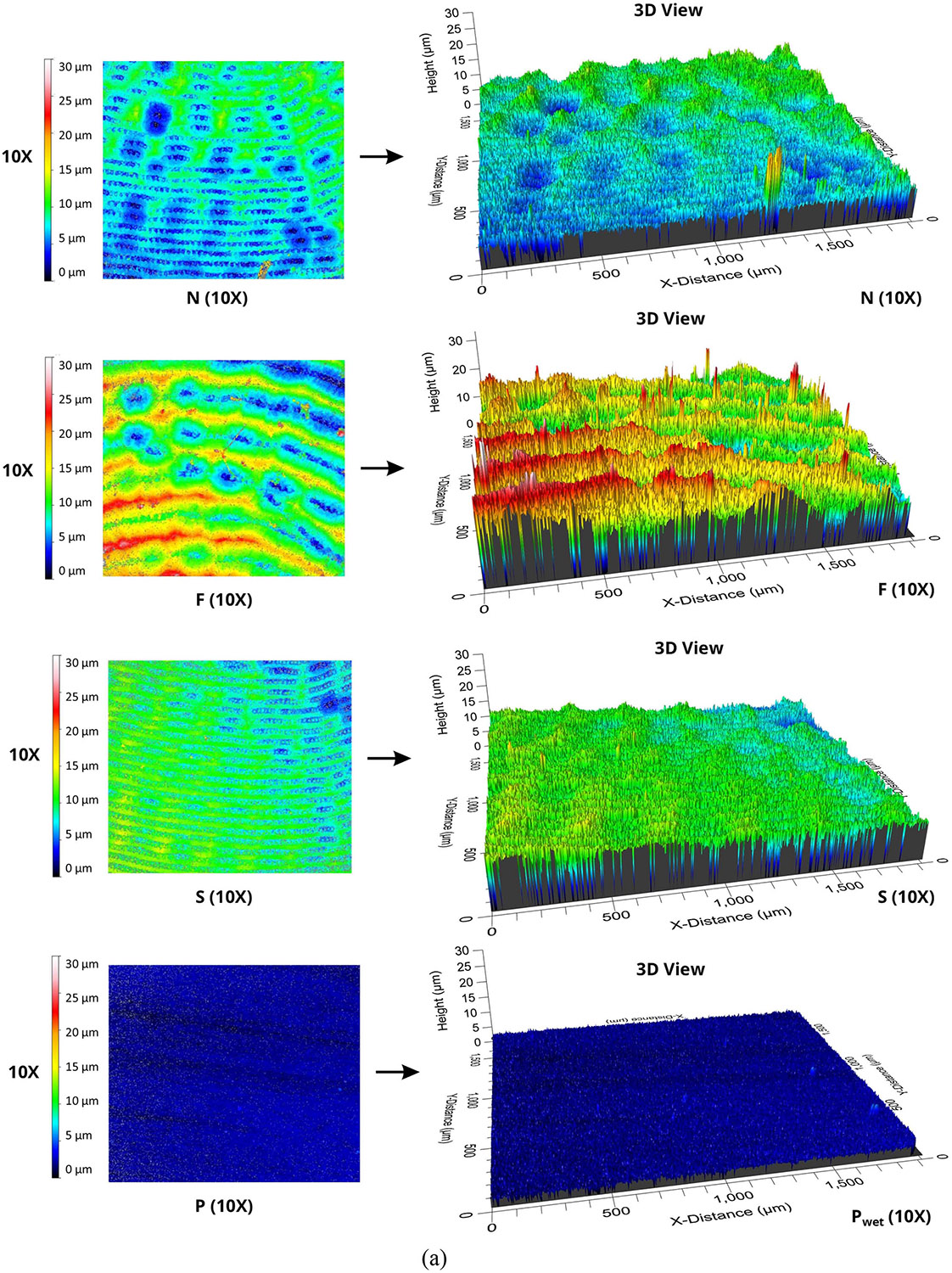

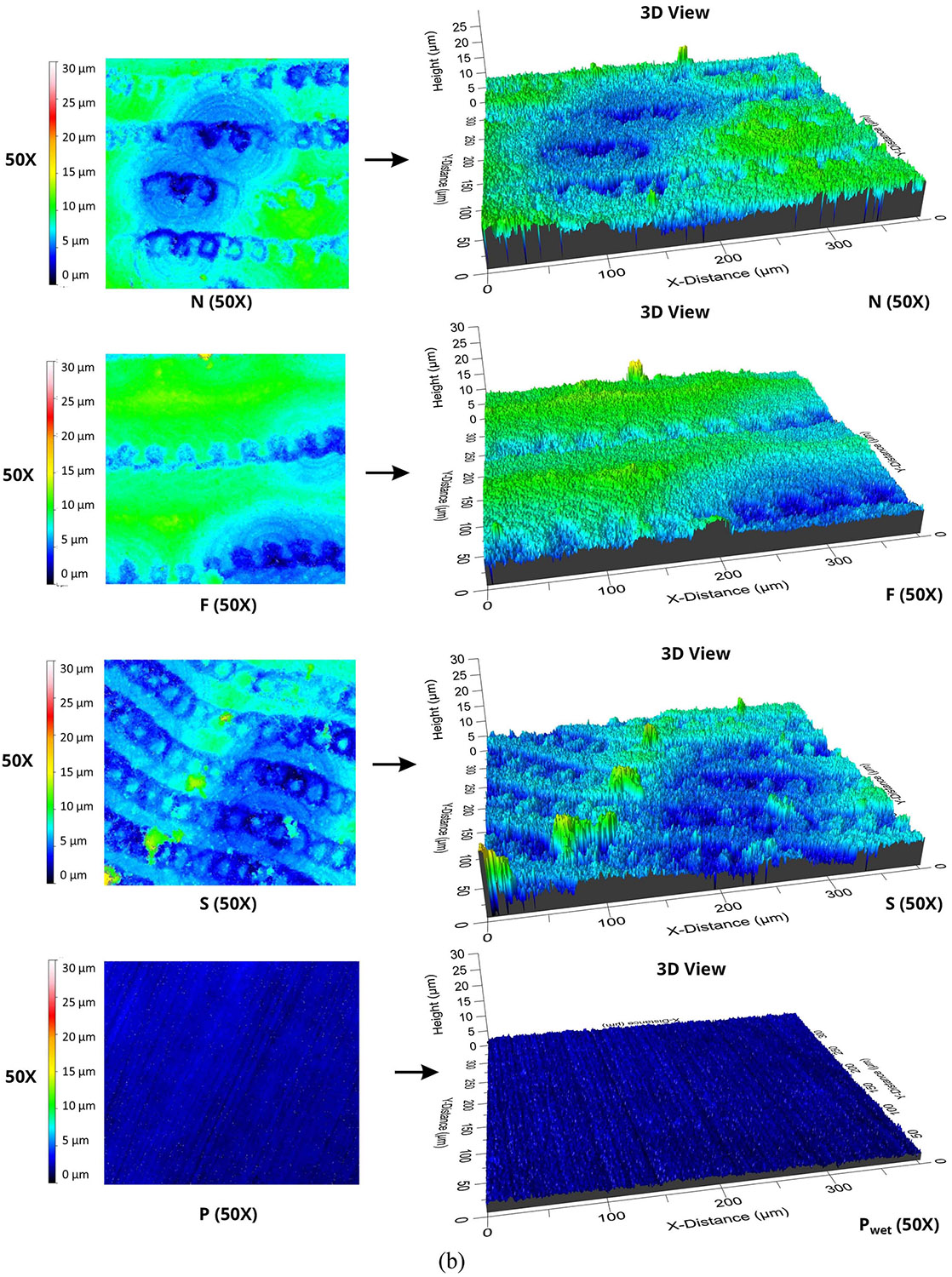
Topography images of the specimens` surface for the experimental groups obtained with the optical profilometer using x10 (a) and x50 (b) magnification. The same scale was applied to all images to enable direct comparisons between groups. Warm colors indicate higher roughness values.

**Fig. 3.**
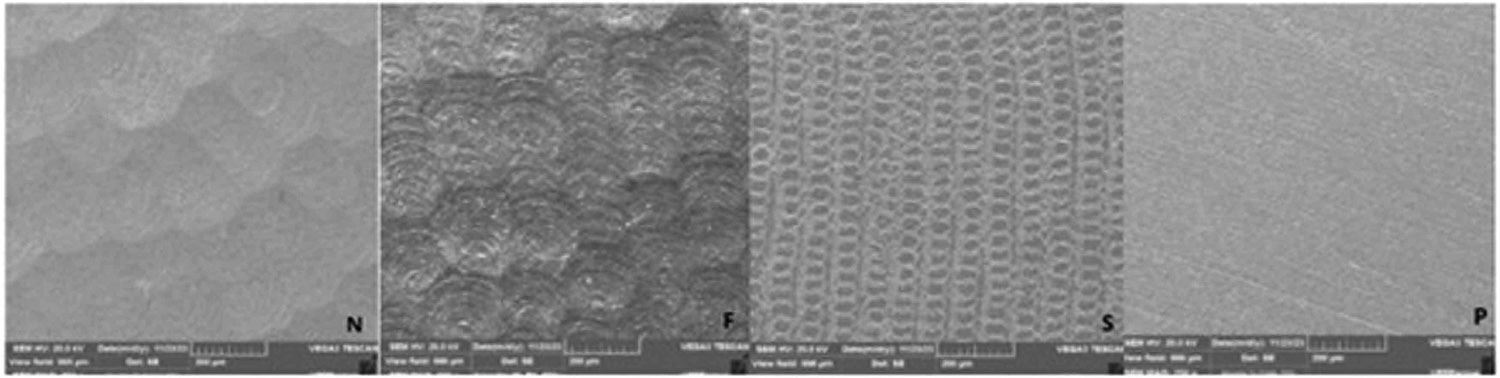
Representative SEM images from specimens` surfaces for the experimental groups (N, F, S, and P (P_wet_)).

Qualitative analysis of the surface topography for the experimental groups obtained using the optical profilometer is presented in Fig. 2. The surface topography of P_wet_ was used to represent both polished groups, as they showed similar surface characteristics. Polished specimens showed a smoother and more homogeneous surface in comparison to the CAD/CAM milled groups. Among the CAD/CAM milled groups, F protocol produced a more heterogeneous surface, with high peaks, in comparison to N and S protocols (Fig. 2a). At a greater magnification, it is possible to observe the different topographies created on the ceramic surface by the paths of the CAD/CAM milling burs (Fig. 2b). Especially for N and S groups, circular paths produced by the fine bur is seen more frequently than in the F group. SEM surface images confirmed the topographic pattern observed using the optical profilometer (Fig. 3).

**Fig. 4.**
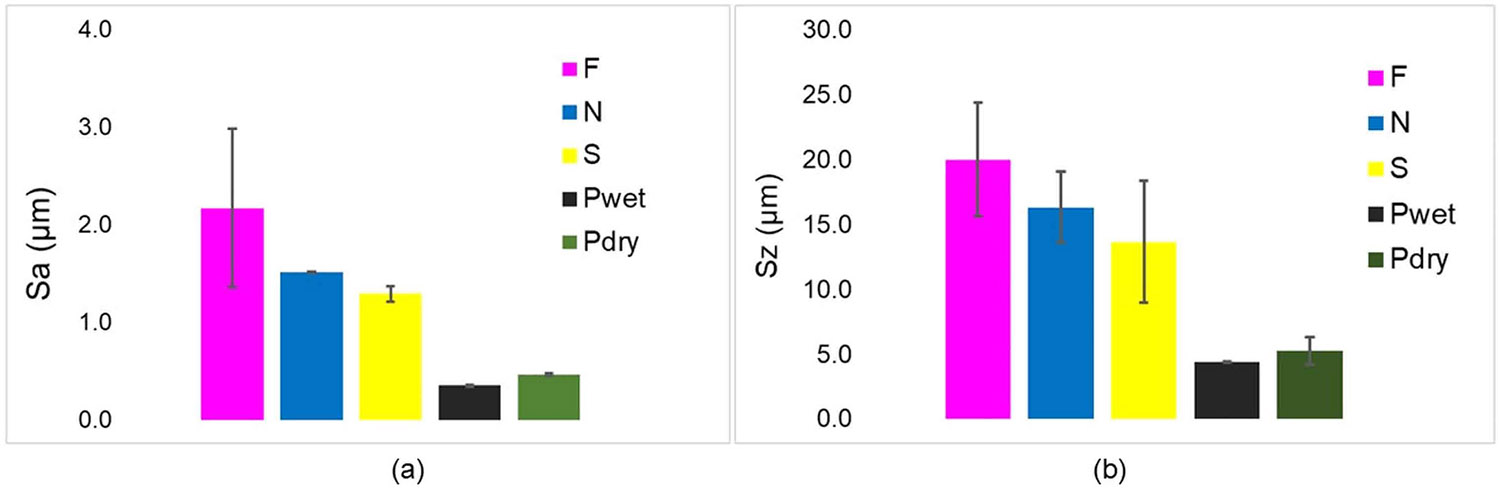
Mean values of Sa (a) and Sz (b) for the experimental groups. Error bars correspond to the standard-deviation values. Descriptive statistical analysis was performed due to the small sample size (n = 3).

Values of the roughness parameters Sa and Sz are presented in Fig. 4. Overall, values followed a decreasing order: F > N > S > P (**P_dry_ and P_wet_**). Descriptive statistical analysis was performed due to the small sample size (n = 3).

### 3.2. Mechanical analysis

**Fig. 5.**
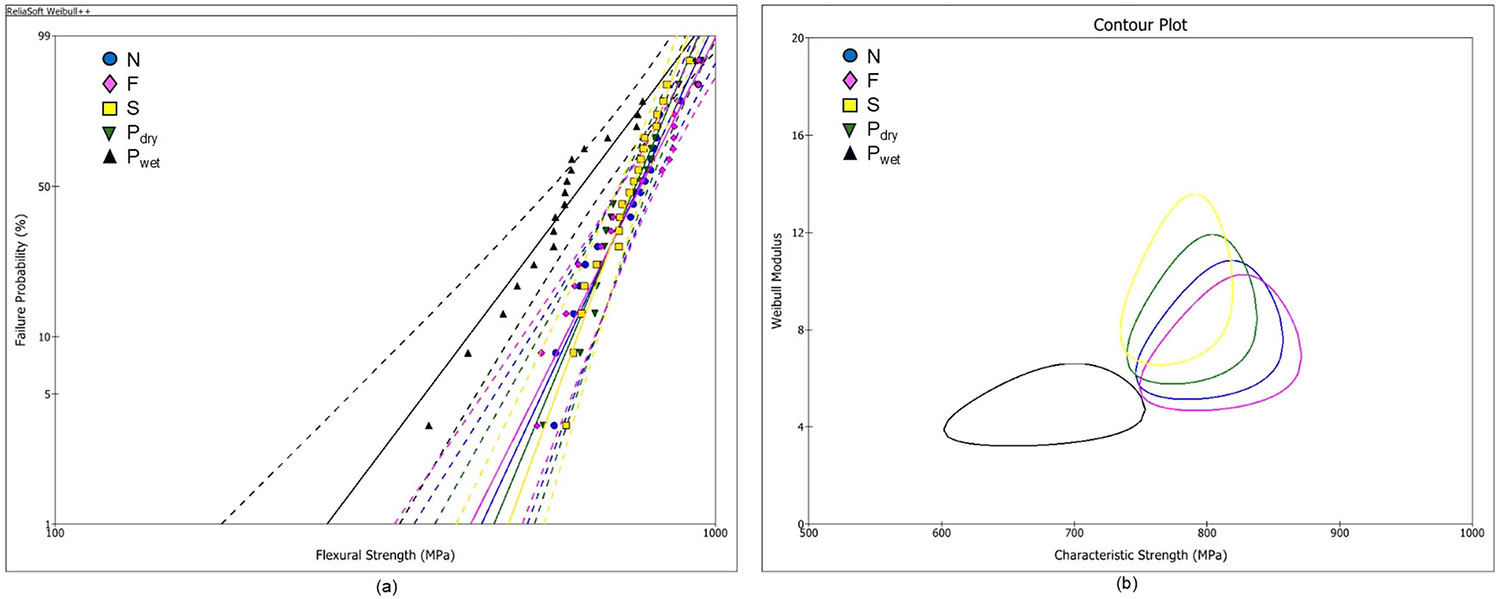
(a) Weibull graph showing the flexural strength data for the tested groups. Doted lines correspond to the 90 % confidence intervals (90 % CI). (b) 90 % CI contour plots for the Weibull modulus (m) and characteristic flexural strength (σ_0_) parameters.

The Weibull plot and analysis parameters for the flexural strength data are presented in Fig. 5a and Table 2, respectively. The chi-square test detected significant differences in the Weibull modulus among groups (χ^2^ = 10.6392; p = 0.031), indicating distinct failure behaviors. Specimens milled with the three types of CAD/CAM protocols and the P_dry_ group showed statistically similar m-values. P_wet_ group exhibited lower m-value, being susceptible to premature failures (k < 1).

The CAD/CAM-milled groups and P_dry_ exhibited similar σ_0_ values, as their 90 % confidence intervals overlapped, whereas P_wet_ showed the lowest σ_0_. A similar trend was observed for the flexural strength values for a 5 % failure probability (Table 2). Fig. 5b shows the 90 % CI contour plot for m and σ_0_ parameters. It can be observed that the confidence region for the P_wet_ group is positioned in the lower-left area of the plot, corresponding to lower values of flexural strength and Weibull modulus.

**Fig. 6.**
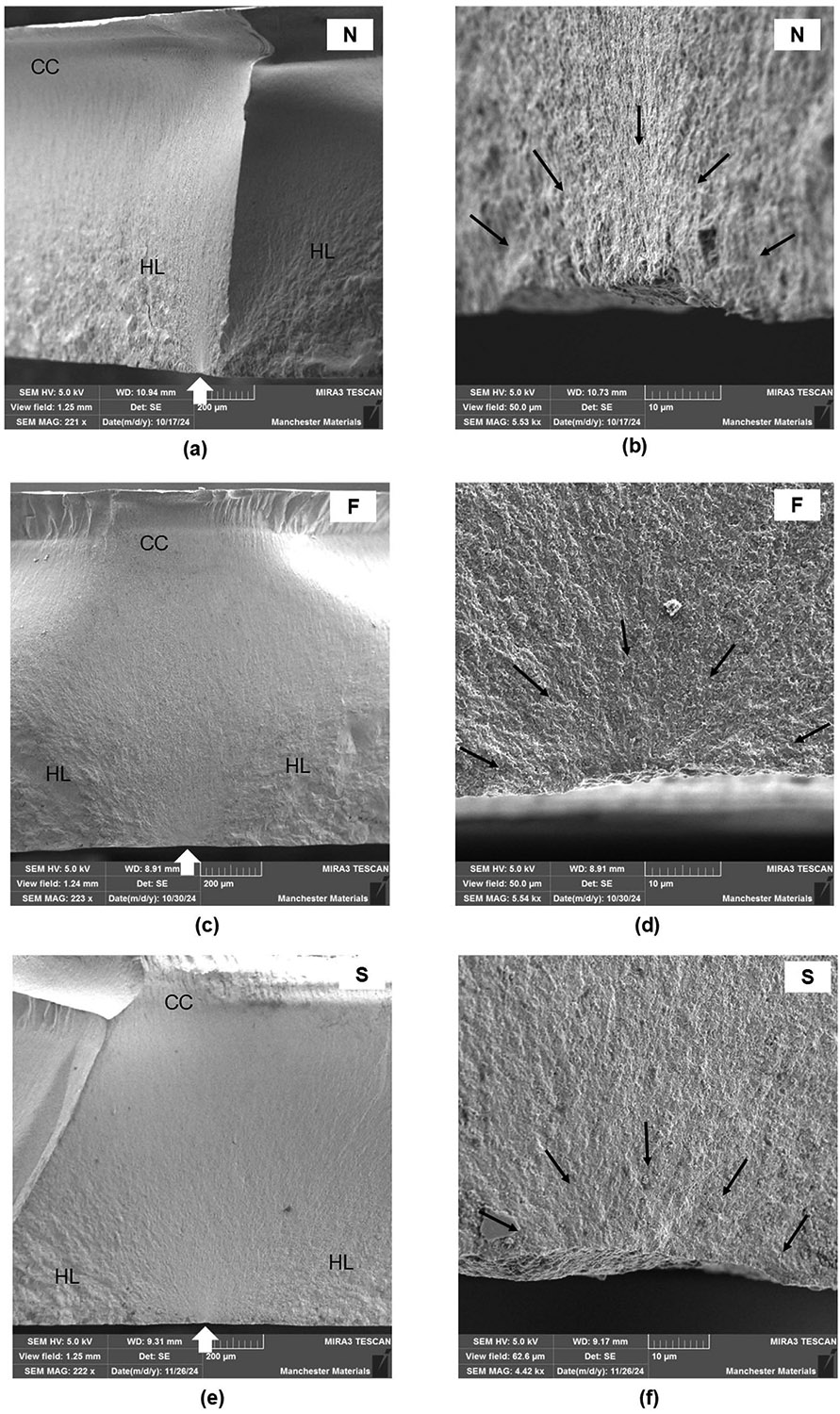
Representative SEM images of the fracture surface of CAD/CAM milled groups: N (a-b), F (c-d), and S (e-f). In images (a), (c), and (e) it is possible to identify a compression curl (CC) and hackle lines (HL) pointing to the crack origin (white arrow) located in the surface of the specimen subjected to tension during the test. The critical flaw is delimited by black arrows in images (b), (d), and (f).

**Fig. 7.**
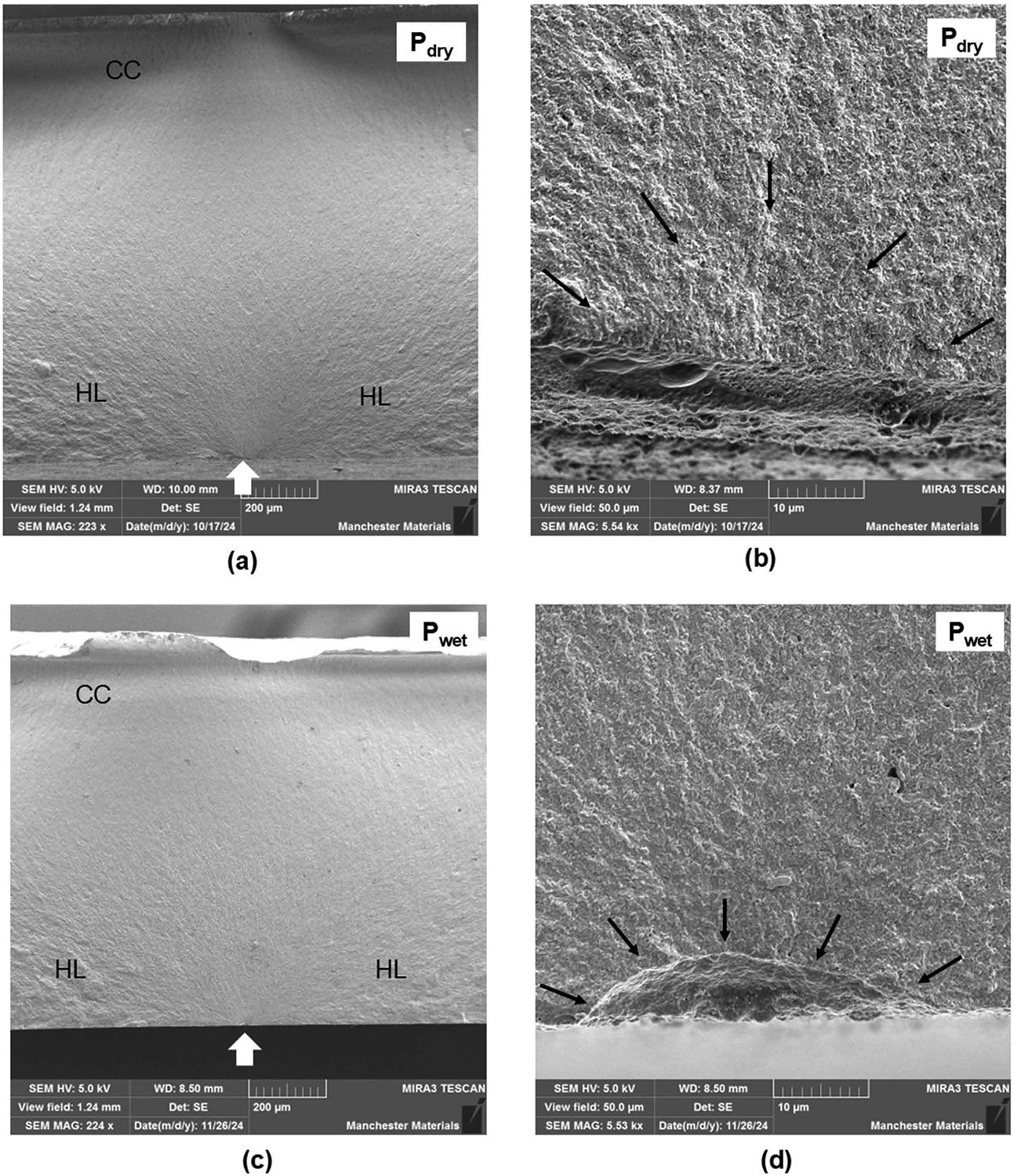
Representative SEM images of the fracture surface of polished groups: P_dry_ (a-b) and P_wet_ (c-d). Compression curl (CC) and hackle lines (HL) pointing to the crack origin (white arrow) are shown in images (a) and (c). The critical flaw is delimited by black arrows in images (b) and (d).

Representative SEM images of the fracture surface of CAD/CAM-milled and polished groups are shown in Figs. 6 and 7, respectively. Similar fractographic features were observed across all experimental groups. In all specimens, it was possible to identify a compression curl and hackle lines pointing to the crack origin located in the surface of the specimen that was loaded in tension.

### 3.3. Crystal phase identification

**Fig. 8.**
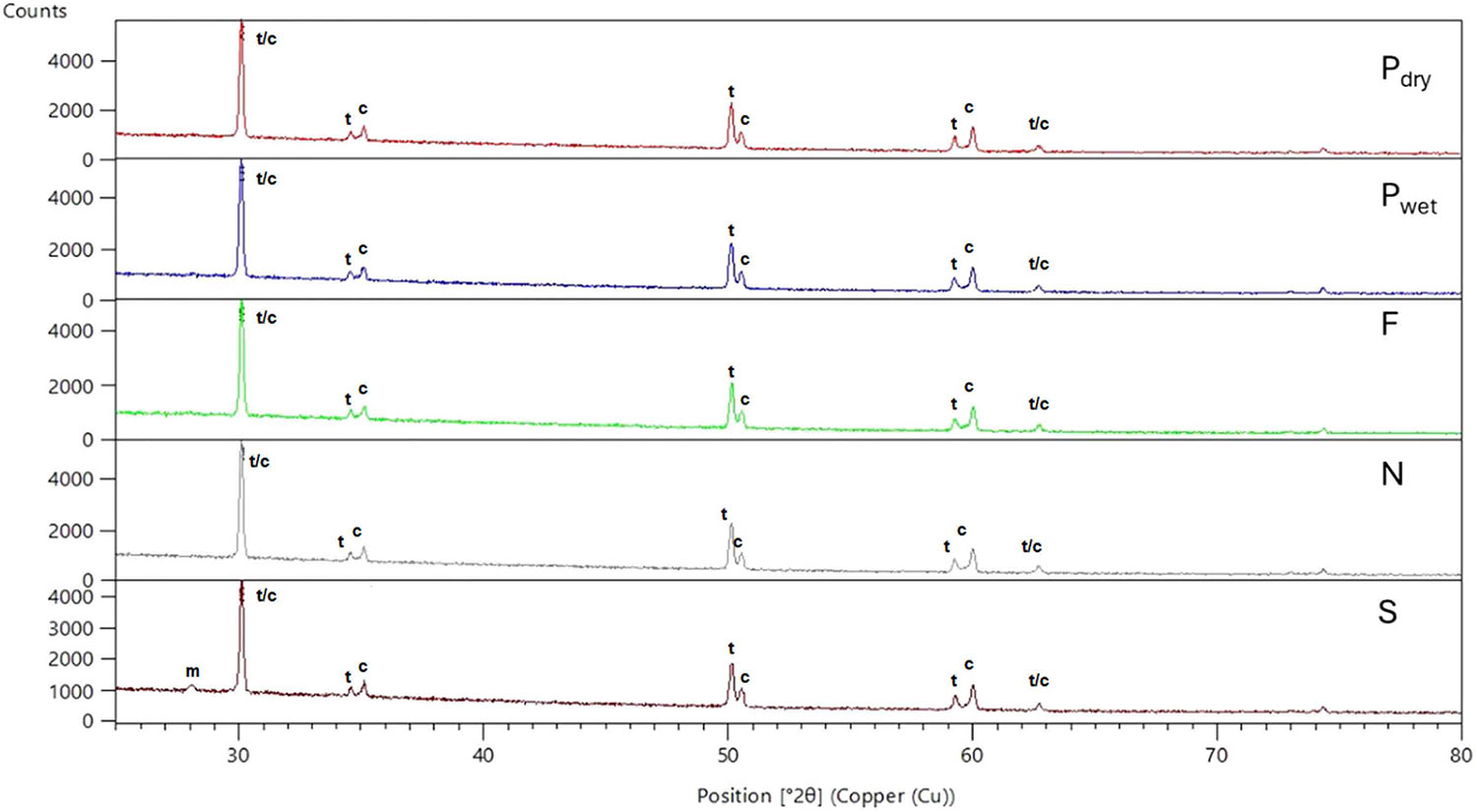
XRD pattern showing the identification of crystalline phases for the experimental groups.

All experimental groups showed similar diffractogram patterns, predominantly featuring tetragonal (81–86 %) and cubic (13–14 %) crystal phases (Fig. 8). Only a small monoclinic peak was observed in group S (<6 %).

## 4. Discussion

As novel CAD/CAM protocols were developed to produce more personalized dental treatments, new variables were introduced that could affect the quality of the final restoration [9,10,15-17]. In the present investigation, the type of CAD/CAM milling protocol influenced on the surface topography pattern of the 3Y-PSZ specimens, while the flexural strength and Weibull modulus were not affected, partially accepting the first study hypothesis.

In the present study, a laboratory 5-axis milling unit with automatic bur control was used to dry-mill the pre-sintered 3Y-PSZ specimens using special diamond-coated burs. In this scenario, a monolithic zirconia premolar crown can be milled in 25 min, 18 min and 12 min using the slow, normal and fast protocols, respectively [9]. The three milling protocols differ in speed and bur milling path. The fast protocol led to a reduced machining time but produced a rougher and more heterogeneous surface compared to the other two CAD/CAM protocols. Overall, the normal and slow protocols resulted in similar surface topography, showing small circular marks produced by the fine bur. Yet, as longer time was required to produce specimens with the slow protocol, the surface seems slightly more homogeneous and smoother than the ones produced with the normal protocol. Similar results were reported in recent studies, showing that the fast milling produces a more heterogeneous surface topography compared to slower protocols [9,10].

Nevertheless, despite the variations in surface topography, the reliability of 3Y-PSZ, represented by the characteristic flexural strength and Weibull modulus, was similar among CAD/CAM milled groups. These findings suggest that the CAD/CAM milled groups have a similar flaw distribution, and the tested protocols did not introduce additional surface defects capable of compromising the specimens’ fracture strength. Greater surface roughness does not necessarily lead to lower fracture strength. In brittle materials, the size, shape, and location of the critical flaw are more important than surface roughness itself [19]. Fractographic analysis showed similar fracture behavior among the CAD/CAM milled groups, with the critical flaw located in the specimen surface subjected to tensile stresses during loading.

On the contrary, an investigation concluded that the fast protocol produces 3Y-PSZ monolithic crowns with higher fracture load after mechanical aging than the normal and slow protocols [9]. The authors attributed these results to differences in the crown geometry, as a rougher and less refined anatomy produced by the fast protocol created a thicker occlusal surface. Moreover, the study [9] found a greater reliability for crowns produced with the slow protocol. In the present study, S-group also exhibited a high value of Weibull modulus resulting in high flexural strength for a 5 % failure probability. A high Weibull modulus suggests greater structural reliability due to less variability in the flexural strength data and higher precision in the probability of failure predictions.

As previous studies have reported a deleterious effect of CAD/CAM milling on the mechanical properties of dental ceramics [11,13], a polished dry-processed control group (P_dry_) was investigated. Despite the reduction in roughness, P_dry_ showed similar flexural strength and Weibull modulus than the CAD/CAM-milled groups. These results could be attributed to the fact that the different surface treatments, CAD/CAM milling and polishing, were performed with 3Y-PSZ in a pre-sintered state. The sintering process enhances the ceramic density and toughness; it is capable of sealing surface defects and releasing residual stresses induced during processing [20]. Processing zirconia in a pre-sintered state is also desired as it does not lead to major alterations in the crystal phase distribution, as observed in the XRD patterns for the different experimental groups. All groups exhibited diffraction peaks corresponding to tetragonal and cubic phases, with intensities comparable to those reported by Vardhaman et al. [8]. A small monoclinic peak was observed in group S (<6 %); however, this should be interpreted with caution because the results are semi-quantitative. Moreover, tetragonal-to-monoclinic phase transformation is usually induced when polycrystalline zirconia is processed post-sintering, and its effect of the material’s long-term mechanical behavior is controversial [3,4].

Another variable of the CAD/CAM machining process refers to the milling environment, which can be dry or wet [15-17]. Therefore, a P_wet_ group was evaluated aiming to further investigate the effect of wet-processing on the mechanical behavior of 3Y-PSZ. Unlike the P_dry_ group, P_wet_ showed the lowest characteristic strength and Weibull modulus among all experimental groups, rejecting the second study hypothesis. Overall, the presence of water during the preparation of polished pre-sintered specimens had a negative impact on 3Y-PSZ mechanical properties. According to Anand *et al.* (2018) [17], grinding pre-sintered zirconia in wet conditions result in greater sub-surface damage and loss of strength compared to dry processing. The presence of water molecules leads to the weakening of interparticle bonding strength and the formation of voids. The low Weibull modulus observed for the P_wet_ group indicates a more heterogeneous flaw population and premature failures, suggesting that the sintering protocol was unable to fully eliminate these defects. Similarly to our results, Kown *et al.* (2023) [15] did not observe changes in the crystalline phase of polycrystalline zirconia milled under dry and wet conditions. Yet, their investigation concluded that wet-milled specimens had greater mechanical properties than dry-milled ones, which was mainly attributed to differences in the materials’ density. Nevertheless, direct comparison between studies is limited by differences in the materials composition and processing conditions. Additionally, in the present study, only polished specimens were investigated under wet and dry conditions. Future studies should investigate in depth the effect of wet-CAD/CAM milling on polycrystalline zirconia properties.

Clinically, additional treatments are often required to either produce smother, more homogeneous external surfaces on CAD/CAM-milled restorations or to create micromechanical retention at the cementation surface for bonding [21,22]. Although different milling strategies may produce slight variations in roughness, clinical evidence suggests that the surface pre-treatment step plays a more decisive role in adhesive retention and long-term success of polycrystalline zirconia [23].

Recent studies indicate that the ideal surface roughness for zirconia abutments in areas of soft tissue contact is approximately 0.2 μm, which is effective in preventing bacterial plaque and promoting tissue attachment [24]. Clinical investigations showed that polished zirconia causes less wear on the opposing enamel than glazed zirconia, but these differences decrease over time. Polished zirconia also induces antagonist wear comparable to enamel [25-27]. However, there is no clear consensus on a direct correlation between surface roughness and wear, especially due to the great number of variables involved in the wear process and high methodological variability [20,25,28,29]. Thus, minor differences observed in surface quality between N and S groups may not affect their wear behavior. Even so, maintaining low roughness remains important for patient comfort. In this context, CAD/CAM dry-milling with a slower protocol could be recommended to reduce the number of processing steps and produce 3Y-PSZ monolithic restorations with high-quality surface finish, without compromising the materials’ structural integrity. This is particularly relevant because polishing the complex geometry of polycrystalline zirconia prostheses is challenging, especially after sintering.

Standard disc-shaped specimens with flat surfaces were evaluated aiming to reduce the number of research variables. The milling bur pattern created in the specimens’ surface simulated the milled internal surface of a crown, where critical flaws that lead to clinical failures are frequently located [4,9,19]. However, the complex configuration of a dental restoration and the effect of cyclic loading on the materials’ mechanical properties were neglected, which are study limitations. Extrapolation of the study findings should also consider that only one type of CAD/CAM system and polycrystalline zirconia brand were investigated. Furthermore, a larger sample size is desired for a more accurate topographic analysis.

## 5. Conclusions

Distinct CAD/CAM dry-milling protocols were able to produce different surface topographies in 3Y-PSZ, while preserving its mechanical properties and crystal phase contents. In addition, drypolished pre-sintered 3Y-PSZ specimens showed superior mechanical behavior than wet-polished ones; further work is needed with wet-milled groups.

## Figures and Tables

**Table 1:** Description of the experimental groups.

Legend	Protocol	Processing	Dry/ Wet
N	Normal	CAD/CAM milling	Dry
F	Fast	CAD/CAM milling	Dry
S	Slow	CAD/CAM milling	Dry
P_dry_	Polished dry (Control)	Cutting machine + manual polishing (pre-sintered state)	Dry
P_wet_	Polished wet	Cutting machine + manual polishing (pre-sintered state)	Wet

**Table 2:** Values of characteristic strength (σ_0_), Weibull modulus (*m*), and flexural strength for a 5 % failure probability (σ_5 %_) with their respective 90 % confidence intervals (90 % CI) for the experimental groups.

Groups	σ_0_* (MPa)	σ_0_ – 90 % CI (MPa)	m**	m - 90 % CI	σ_5 %_* (MPa)	σ_5 %_ - 90 % CI (MPa)
N	801.4 a	760.0; 843.1	7.7 ab	5.7; 10.1	545.7 a	463.3; 611.2
F	809.7 a	764.1; 855.3	7.2 ab	5.2; 9.5	534.5 a	444.6; 606.5
S	777.1 a	745.1; 808.9	9.8 a	7.3; 12.6	573.6 a	505.4; 626.5
P_dry_	788.4 a	751.4; 825.2	8.6 a	6.4; 11.1	558.0 a	483.3; 616.8
P_wet_	675.4 b	619.5; 733.2	4.8 b	3.6; 6.2	362.7 b	280.6; 434.2

* Values followed by the same letter in the same column are similar as the 90 % CI overlap.

** Weibull modulus was statistically analyzed with chi-square test (χ^2^ = 10.6392; p = 0.031) and Bonferroni-adjusted 90 % simultaneous confidence intervals were used for multiple pairwise comparisons.
